# Role of Lymph Node Dissection in Commonly Diagnosed Solid Organ Malignancies With High Mortality Rates: A Systematic Review and Meta-Analysis of Randomized Controlled Trials

**DOI:** 10.7759/cureus.88981

**Published:** 2025-07-29

**Authors:** Soroush Bazargani, Srivani Sanikommu, Seyed Behzad Jazayeri, Mohammed Al-Toubat, Daniel Norez, Navin Balaji, Aditya Sathe, Gretchen Kuntz, Shiva Gautam, K.C. Balaji

**Affiliations:** 1 Urology, University of Florida College of Medicine – Jacksonville, Jacksonville, USA

**Keywords:** bladder cancer, breast cancer, endometrial cancer, liver cancer, lung cancer, lymph node dissection, prostate cancer, randomized controlled trial, solid-organ malignancies, survival

## Abstract

The purpose of this study is to review the literature and compare the outcomes of lymph node dissection (LND), or lymphadenectomy, versus no lymphadenectomy (no LND) and extended lymphadenectomy (ELND) versus standard lymphadenectomy (SLND) in various commonly diagnosed solid malignancies with high mortality rates in the United States. We searched for randomized controlled trials involving high-mortality solid tumors, including prostate, bladder, lung, breast, colorectal, pancreas, liver, endometrial, ovarian, and esophageal cancers, in Medline, Embase, and Cochrane Library. The primary endpoint was overall survival, and secondary endpoints included progression-free survival and complications. We identified 4,436 studies in a database search. Of the 43 eligible clinical trials in this study, 31 studies (72.1%) compared LND versus no LND, and the remaining 12 studies (27.9%) compared ELND versus SLND. None of the studies in either comparative group showed a significant difference in overall or recurrence-free survival, with the exception of one study in breast cancer, where ELND was associated with improved overall survival (HR 1.15, 95% CI 1.01-1.30; p = 0.03) compared to SLND. However, ELND across several cancers may be associated with increased risks of complications compared to SLND. The results from this study could help with counseling patients regarding the role of LND in staging of the disease, rather than improving the outcomes. Despite the heterogeneity of the cancers included in this study, the commonality of the lack of survival benefit of LND in most cancers identifies an opportunity for further understanding and the need for additional research on the impact of metastasis to lymph nodes on cancer outcomes.

## Introduction and background

Lymph node dissection (LND) or lymphadenectomy is acknowledged for staging and prognostication of most solid tumors; however, its therapeutic role and optimal extent of dissection in each specific cancer have remained debatable to date. Multiple retrospective and prospective studies and randomized controlled trials (RCTs) have been conducted to compare a standard LND (SLND) with extended LND (ELND) based on oncological outcomes [1-5]. Some studies have reported better staging, lower disease recurrence, and improved prognosis with a greater number of lymph nodes analyzed. However, the therapeutic benefits of ELND need to be determined. In some cases, like breast cancer, RCTs comparing either LND versus observation, or ELND versus SLND, have failed to show survival differences between treatment arms [3,4]. 

There is strong and evolving evidence that, despite different organs of origin, human solid tumors share key molecular and genetic mechanisms that influence disease progression and clinical outcomes. [6] These include activation of epithelial-mesenchymal transition (EMT), lymphangiogenesis mediated by VEGF-C and VEGF-D signaling, and expression of chemokine receptors such as CCR7 and CXCR4 that facilitate homing of tumor cells to lymphatic tissue. In addition, mutations in genes such as TP53, KRAS, and PI3K pathway components are frequently observed across multiple tumor types and are associated with increased metastatic potential, including lymphatic spread [7]. Given that lymph node involvement is often a critical step in systemic dissemination, LND has traditionally been employed not only for staging but also as a potentially therapeutic intervention. 

By removing microscopic nodal disease that may not be detectable on imaging, LND could theoretically improve oncologic outcomes by preventing further metastasis and guiding the use of adjuvant therapy. This concept has historically underpinned surgical oncology practices in multiple tumor types, including breast, lung, gastric, colorectal, and genitourinary cancers. Several landmark studies and clinical guidelines have endorsed regional lymphadenectomy as a standard component of cancer surgery in appropriately selected patients [1-5]. However, emerging randomized data have cast doubt on its universal therapeutic value, prompting the need for a comprehensive reappraisal across solid tumors with high mortality. 

Several authors have published systematic reviews and meta-analyses on the role and extent of LND in each organ-system malignancy [1-5, 8-10]. However, there is no published comprehensive review or analysis on the role of lymphadenectomy in commonly diagnosed solid cancers associated with high rates of lymph node metastasis and mortality. 

According to the American Cancer Society (ACS) report 2022, the most common solid malignancies include lung, breast, prostate, colorectal, pancreas, liver, ovarian, esophageal, and bladder cancers [11]. The purpose of this study is to review the literature to compare the outcomes of LND versus no LND and ELND versus SLND in various solid tumors with high mortality. Furthermore, this review focuses on the adverse effects and complications of ELND to SLND using measurable variables including estimated blood loss, operative time, length of hospital stays, and postoperative complications.

## Review

Methods 

Search Strategy

We obtained the list of the most common and highest mortality solid tumors from ACS 2022 Information, and the selected cancer types include lung, breast, prostate, colorectal, pancreas, liver, ovarian, esophageal, endometrial, and bladder [11]. A systematic literature search of electronic databases including Medline, Embase, and Cochrane Library was conducted from inception till May 2022 to identify relevant randomized controlled trials with only the primary end point of overall survival or disease-free survival. To search comprehensively, we have used the following keywords: "lymph node", "lymphadenectomy", "lymph node dissection or excision", "randomized controlled trial", "crossover", and "double-blind" for each relevant cancer (search strategy: Appendix 1). Relevant articles were identified, and the reference lists were also searched. Only full-text articles available in English were included in the review, while retracted articles and prospective trials were excluded.

Study Selection 

We extracted definitions of standard versus extended LND (SLND vs. ELND) as specified in each included trial and tabulated them by cancer type (Table 1). In several studies, however, the exact lymph node counts were not consistently reported; therefore, we were not able to include this metric in our analysis. In addition, we included studies with mixed-stage populations only when stratified outcomes were reported or when the surgical intervention was applied uniformly across comparable clinical contexts (e.g., localized disease). Studies with substantial stage heterogeneity or unclear staging criteria were evaluated carefully for bias.

**Table 1:** Definitions of SLND vs. ELND by cancer type SLND: standard lymphadenectomy, ELND: extended lymphadenectomy

Cancer type	Standard LND (SLND)	Extended LND (ELND)	Avg. nodes removed (if available)
Breast	Sentinel lymph node biopsy (SLNB; level I)	Axillary lymph node dissection (levels I–III)	SLND: 1–4; ELND: 10–20
Lung	Hilar or selective mediastinal sampling	Complete mediastinal dissection (multiple N2 stations)	SLND: ~7; ELND: ~12–15
Prostate	Limited pelvic (obturator, external iliac)	Extended pelvic (obturator, external/internal iliac, presacral, and common iliac nodes)	SLND: 8–10; ELND: 20–28
Bladder	Standard pelvic (obturator and external iliac)	Extended (includes internal iliac, presacral, and common iliac)	SLND: 12–16; ELND: 20–30
Colorectal	Mesorectal excision (central lymphadenectomy)	High ligation of inferior mesenteric artery with extended mesenteric clearance	Not consistently reported
Pancreas	Peripancreatic LND	Extended to celiac, para-aortic, and hepatoduodenal ligament	SLND: ~11–15; ELND: ~18–25
Liver	Perihepatic or hilar nodes	Additional peripancreatic and celiac dissection	Limited data available
Endometrial	Pelvic node dissection	Pelvic + para-aortic node dissection	SLND: 10–12; ELND: 20–30
Ovarian	Pelvic or selective para-aortic	Systematic pelvic + para-aortic dissection	ELND: up to 35+
Esophageal	Lower mediastinal or upper abdominal only	Two- or three-field dissection (cervical, thoracic, abdominal)	Variable; not always reported

Our inclusion criteria were as follows: (1) prospective randomized studies; (2) studies comparing LND versus no LND; (3) studies comparing SLND to ELND; (4) outcome being represented in OS or RFS; (5) demographics and pathologic characteristics of patients provided; (6) secondary endpoints like estimated blood loss, operative time, and complications provided. 

Exclusion criteria were either of the following: (1) retrospective studies, (2) prospective non-randomized trials, (3) systematic reviews, (4) retracted articles, (5) no available oncological outcomes, (6) inability to obtain full-text, or (7) non-English language.

Data Extraction 

Two reviewers independently screened and extracted data from the included studies using a standardized Excel spreadsheet. Extracted items included the primary author’s name, study design, country of origin, patient characteristics, follow-up duration, and reported outcomes. Discrepancies between reviewers were resolved through discussion or consultation with a third reviewer. In cases of missing or unclear data, study authors were contacted for clarification. Studies were excluded if no response was received within the defined timeframe. 

Quality Assessment

The methodological quality of all eligible studies was assessed using the Newcastle-Ottawa Scale (NOS), which evaluates three main domains: selection of study groups, comparability of groups, and ascertainment of outcomes. Each domain criterion was awarded a maximum of one star if adequately addressed. Based on total scores, studies were categorized as having poor (zero to three stars), fair (four to six stars), or good quality (seven to nine stars).

Risk-of-Bias Assessment

The risk of bias for non-randomized studies was further evaluated using the Risk of Bias in Non-randomized Studies of Interventions (ROBINS-I) tool. This instrument assesses bias across several domains, including confounding, participant selection, intervention classification, deviations from intended interventions, missing data, outcome measurement, and selective reporting. Each domain was rated as having low, moderate, or serious risk of bias, and an overall bias judgment was derived for each study accordingly.

Statistical Analysis

Data extraction was performed by two independent researchers. Studies within their respective cancers and with similar outcome measures were pooled for an overall estimated effect with a 95% confidence interval. All cancers and their estimated effects were plotted relative to the null value of 1. This plot is intended to visualize the effect of lymphadenectomy among all cancers, but does not combine estimated effects for an overall measure. All analyses were performed with STATA statistical software (release 15, College Station, TX: StataCorp LLC.).

Outcome Measures 

**Figure 1:**
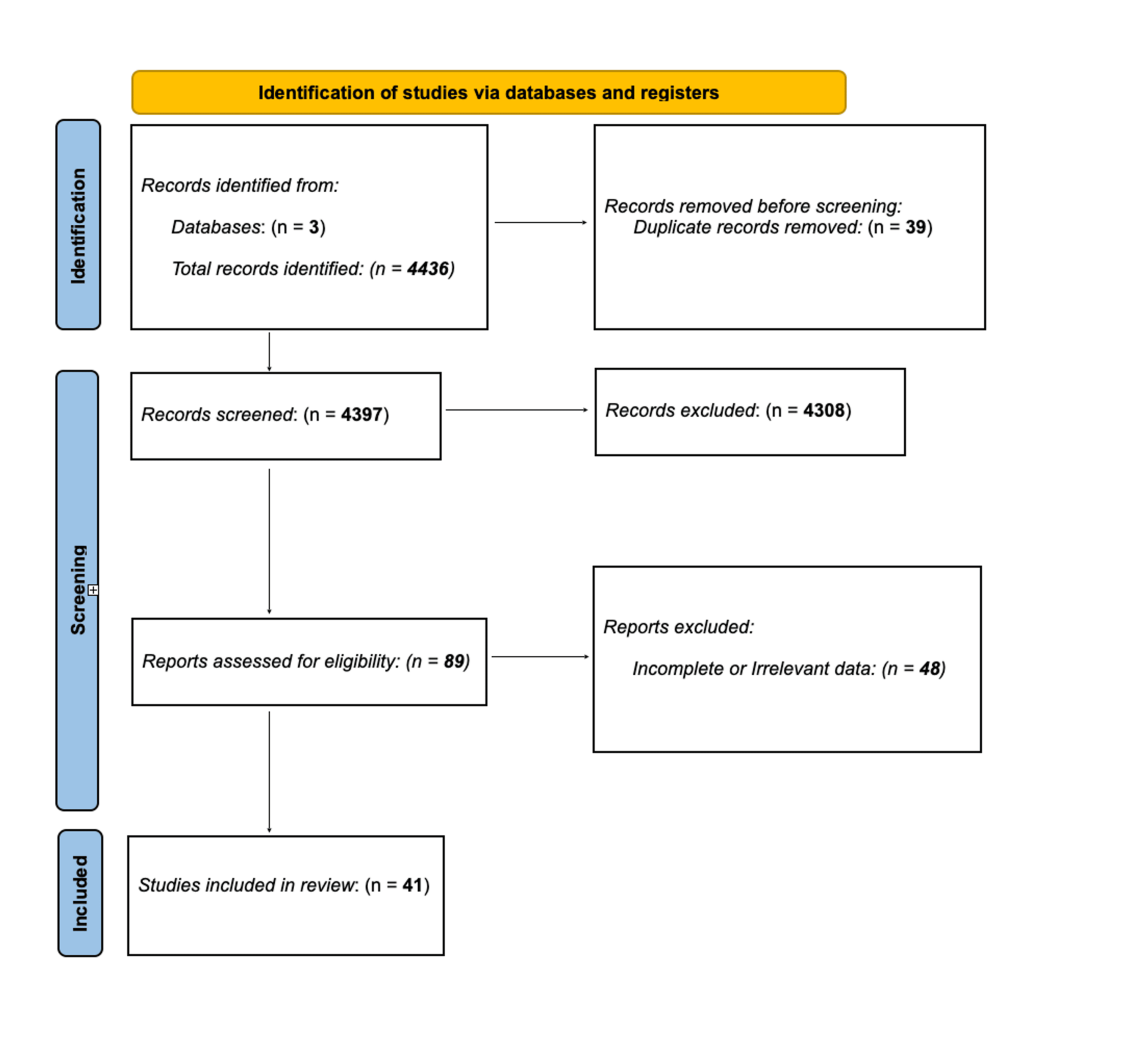
Preferred Reporting Items for Systematic Reviews and Meta-Analysis (PRISMA) flow diagram

The primary outcome was overall survival, and the secondary outcome measures of interest included progression-free survival, adverse events, and complications like estimated blood loss, operative time, and others. The search strategy is shown in Appendix 1, and the Preferred Reporting Items for Systematic Reviews and Meta-Analysis (PRISMA) diagram is summarized in Figure 1. 

Results 

Overview

A total of 4,436 studies were identified through the database search. After removal of 39 duplicates and initial screening of title and abstract by two independent researchers, 4,308 articles were excluded (apparently irrelevant studies, study type of case, series/case reports, letters/reviews/comments). The remaining 89 full-text articles were reviewed in depth for data collection. However, 48 articles did not qualify for the meta-analysis due to prospective non-randomized trials (5), retracted articles (1), and incomplete or irrelevant data (42). These were later used for the systematic review section of the study. Ultimately, 41 full-text articles were included in this meta-analysis following stratification for the relevant cancer type. 

**Figure 2:**
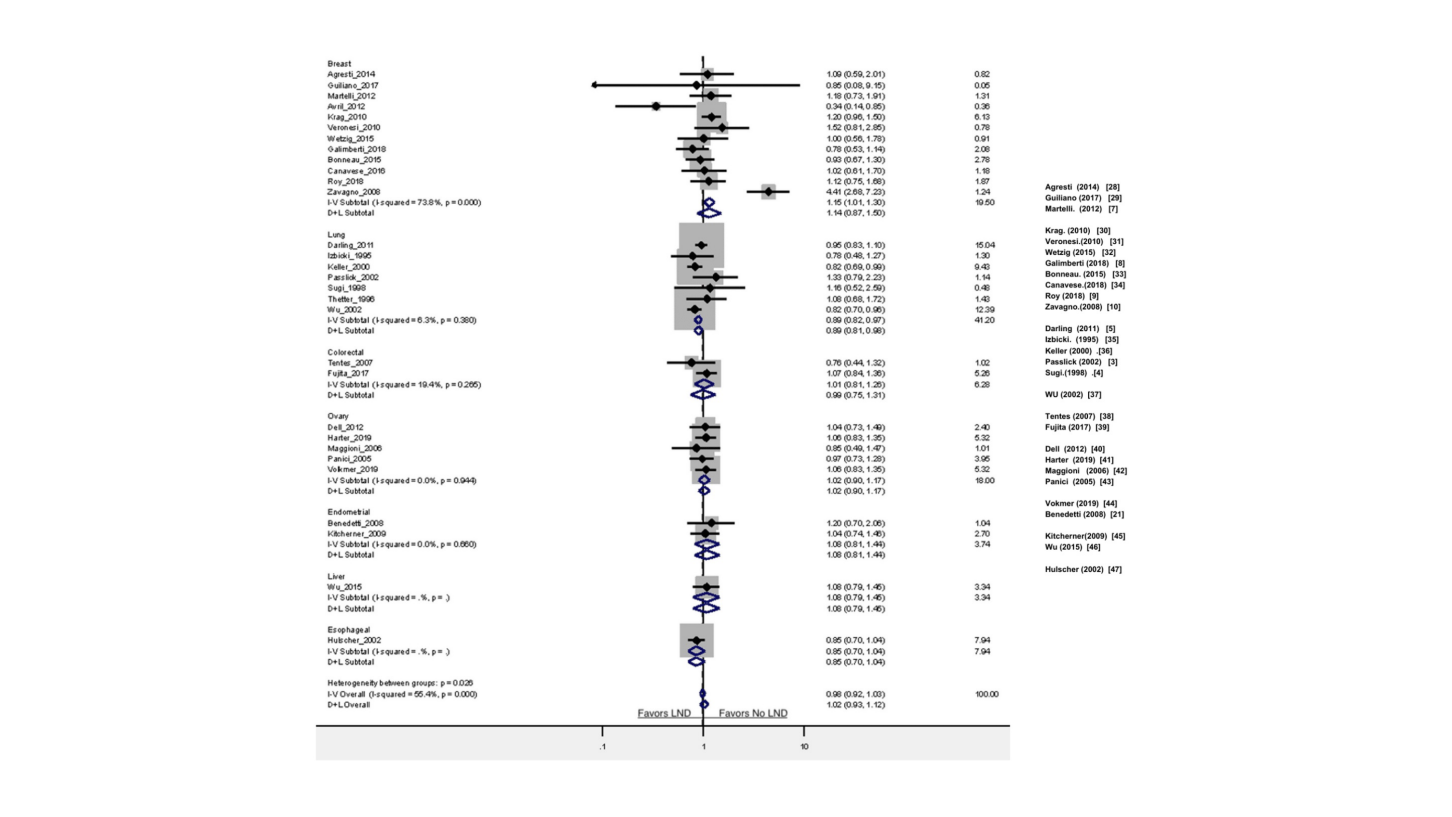
Comparison of LND vs. no LND with regard to overall survival in the available RCTs LND: lymph node dissection, RCT: randomized controlled trial

**Figure 3:**
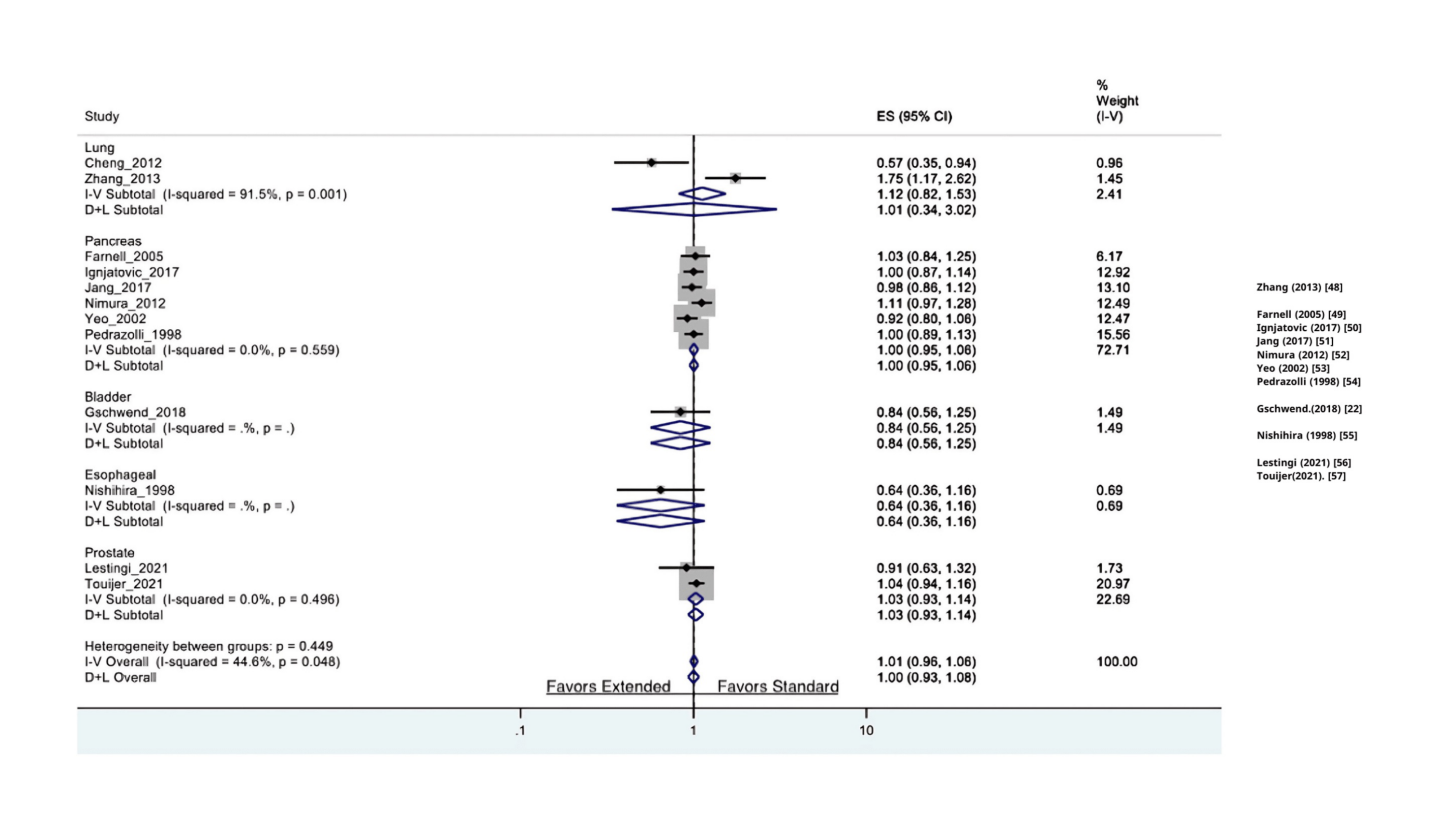
Comparison of extended LND vs. standard LND with regard to overall survival in the available RCTs LND: lymph node dissection, RCT: randomized controlled trial

**Figure 4:**
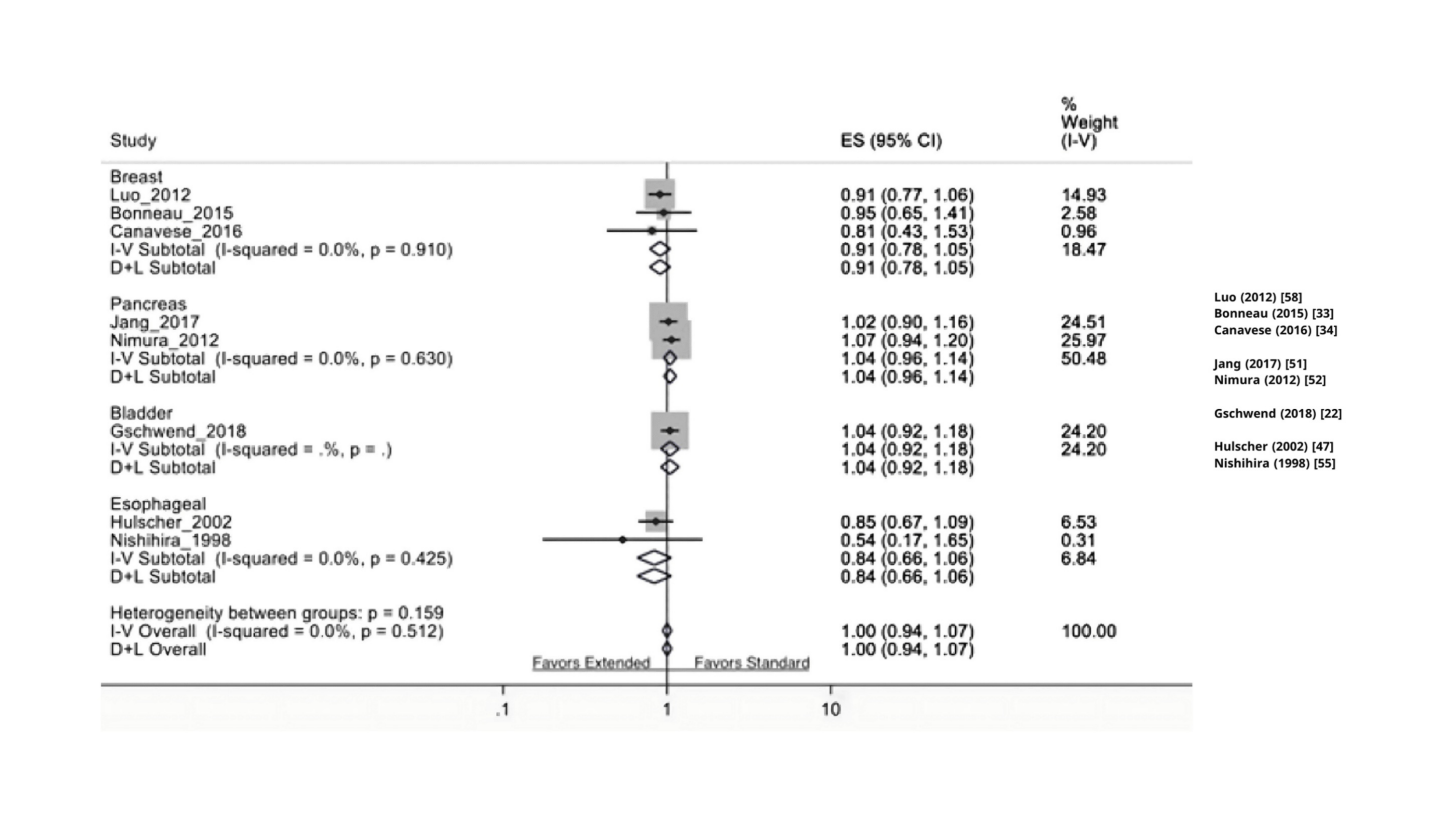
Comparison of extended LND vs. standard LND with regard to recurrence-free survival in the available RCTs LND: lymph node dissection, RCT: randomized controlled trial

**Figure 5:**
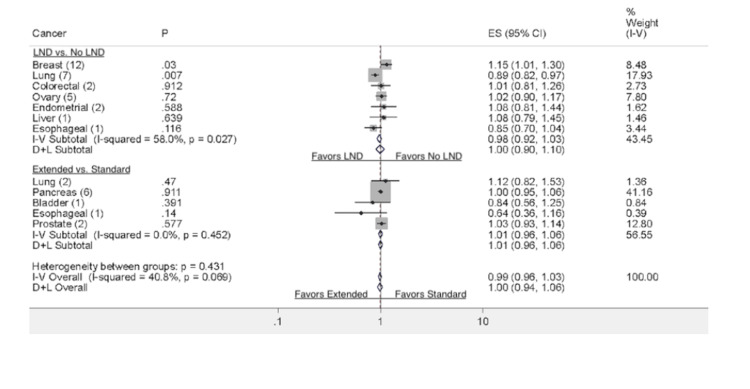
Summary of forest plots comparing LND vs. no LND and ELND vs. SLND with regard to OS in commonly diagnosed genitourinary and other solid organ malignancies. LND: lymph node dissection, ELND: extended lymph node dissection, SLND: standard lymph node dissection, OS: overall survival

**Figure 6:**
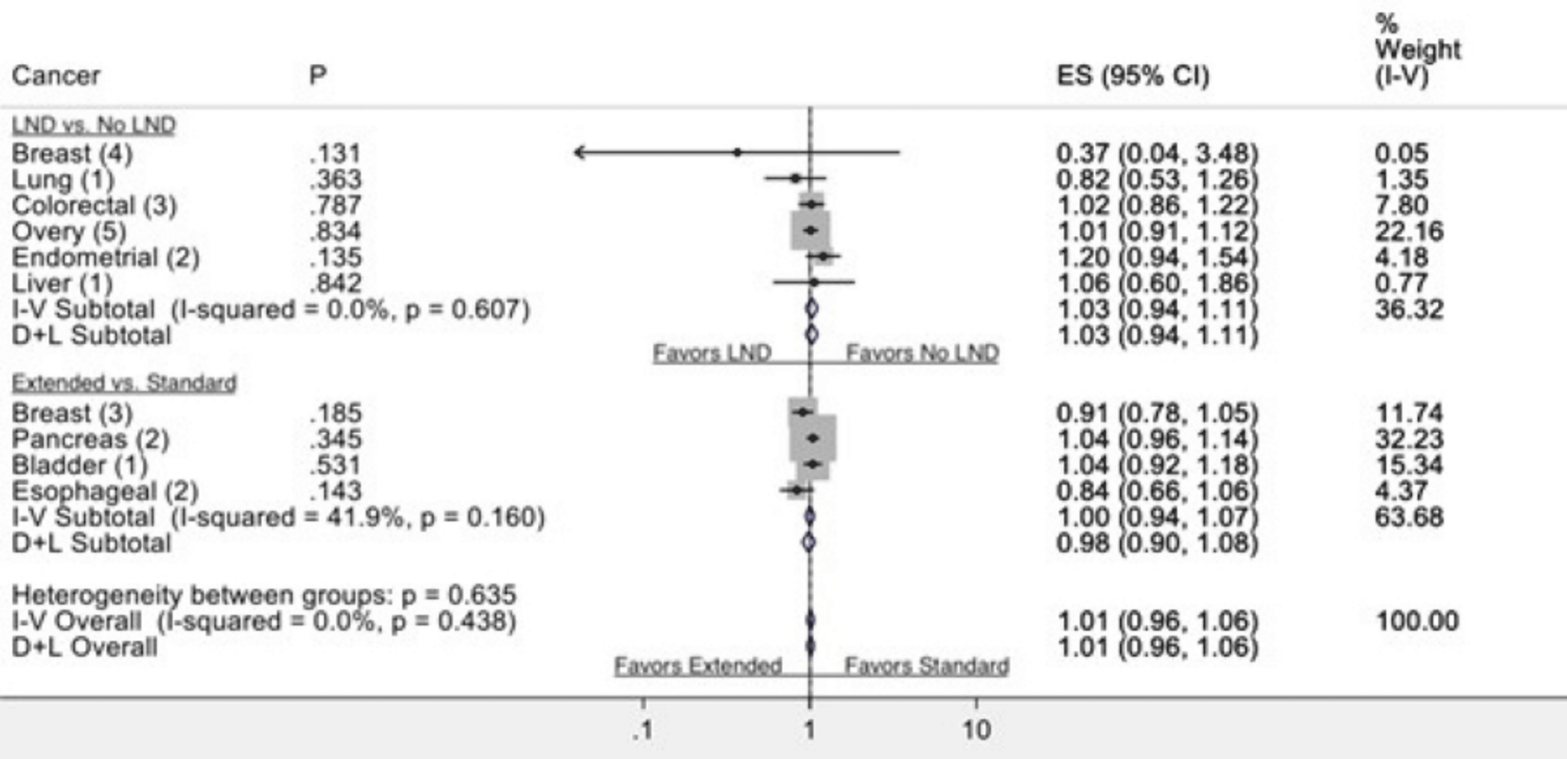
Summary of forest plots comparing LND vs. no LND and ELND vs. SLND with regard to RFS in commonly diagnosed genitourinary and other solid-organ malignancies LND: lymph node dissection, ELND: extended lymph node dissection, SLND: standard lymph node dissection, RFS: recurrence-free survival

In this review, we included eight breast cancer trials comparing axillary LND to no LND, five breast cancer trials comparing ELND (axillary) to SLND (sentinel), seven lung cancer trials comparing ELND (mediastinal) to SLND (hilar), six pancreatic trials comparing ELND to SLND, one RCT about hepatocellular carcinoma comparing LND (hepatectomy) to no LND, two endometrial cancer trials comparing LND to no LND, two colorectal cancer trials comparing LND (mesorectal) to no LND, one bladder cancer study comparing ELND to SLND, two prostate cancer RCTs comparing ELND to SLND, three ovarian cancer RCTs comparing LND (para-aortic) to no LND, and two RCTs in esophageal cancer comparing LND to no LND. The results are depicted in Figures 2-6 and will be described in further detail in subsequent sections below.

Lung Cancer 

There are seven eligible randomized studies comparing LND to no LND in patients with lung cancer. Interestingly, patients who underwent LND compared to those who did not have LND had significantly better overall survival (HR: 0.89 (0.82-0.96 95% CI, p =< 0.05). The I^2^ test (6.3%) revealed no significant heterogeneity. Moreover, patients who received LND versus no LND had no statistical difference based on recurrence-free survival (HR: 0.82 (0.53-1.26 95% CI, P= 0.36)). The data comparing ELND (mediastinal) to patients who had only SLND (hilar) was assessed for overall survival. The two studies by Cheng et al. and Zhang et al. reported on the difference in overall survival between ELND and SLND; the studies found no significant differences between the two groups (HR: 1.12 (0.82-1.53 95% CI, p = 0.001)). The I^2^ test (91.5%) revealed significant heterogeneity amongst the two studies.

Breast Cancer 

Patients who underwent LND compared to those who did not have LND had a significant difference in overall survival (HR: 1.15 (1.01-1.30 95% CI, p= 0.03)). The I^2^ test (17.93) revealed no significant heterogeneity. Moreover, patients who underwent ELND compared to SLND had no significant differences in residual free survival (HR: 0.37 (0.04-3.48 95% CI, p= 0.131)). Furthermore, patients who received LND versus no SLND had no statistical difference based on recurrence-free survival (HR: 0.91 (0.78-1.05 95% CI, p=0.185)). The I^2^ test (0) revealed no significant heterogeneity.

Prostate Cancer 

There are two eligible studies published in prostate cancer to date. The data from Lestingi et al. and Touijer et al., who underwent limited or ELND, were assessed for overall survival. Patients who underwent ELND had no significant differences in overall survival (HR: 1.03 (0.93-1.14 95% CI, p = 0.57)) compared to SLND. The I^2^ test (12.80) revealed no significant heterogeneity

Bladder Cancer 

There is only one eligible study published on bladder cancer at the time of this review. The data from Gschwend et al., who underwent ELND or SLND, were assessed for overall survival. The patients undergoing ELND had no significant differences in overall survival compared to SLND (HR: 0.64 (95% CI 0.36-1.16), p = 0.39). Moreover, patients who underwent ELND had no significant differences in residual-free survival (HR: 1.04 (0.92-1.18, 95% CI, p = 0.53)).

Colorectal Cancer 

The data from two colorectal cancer trials comparing patients who underwent LND (mesorectal) compared to no LND was assessed for overall survival. Patients who underwent LND had no significant differences in overall survival (HR: 1.01 (0.81-1.26 95% CI, p = 0.38)). The I^2^ test (17.93) revealed no significant heterogeneity. Moreover, patients who underwent LND (mesorectal) compared to no LND had no significant differences in residual free survival (HR: 1.02 (0.86-1.22 95% CI, p = 0.78)). 

Pancreatic Cancer

Patients who underwent SLND had no significant differences in overall survival (HR: 1.00 (0.95-1.06 95% CI, p = 0.91)). In the studies from Jang et al. and Nimura et al. that looked at residual-free survival, no significant differences were found between the two groups (HR: 1.04 (0.96-1.14 95% CI, p = 0.63)). The I^2^ test (0%) revealed no significant heterogeneity among the two studies. 

Hepatic Cancer 

Patients who underwent LND (hepatectomy) compared to no LND had no significant differences in overall survival (HR: 1.08 (0.79-1.45 95% CI, p = 0.64)). The I^2^ test (7.8%) revealed no significant heterogeneity amongst the studies included. Moreover, the study found no significant differences in residual free survival between the two groups (HR: 1.06 (0.60-1.86 95% CI, p = 0.84)). 

Ovarian Cancer 

Reviewing studies on ovarian cancer, we found five clinical trials comparing the effect of LND compared to no LND on overall survival. Based on the outcomes of these studies, patients who underwent LND had no significant differences in overall survival compared to those who did not undergo LND (HR: 1.02 (0.90-1.17 95% CI, p = 0.72)). The I^2^ test (7.8%) revealed no significant heterogeneity amongst the studies included. No significant differences were found between LND and no LND with regards to recurrence-free survival (HR: 1.01 (0.91-1.12 95% CI, p = 0.834)). The I^2^ test (22.16%) revealed no significant heterogeneity among the two studies. 

Endometrial Cancer

The data from two endometrial cancer trials comparing patients comparing LND compared to no LND was assessed for overall survival. Based on the outcomes of the two studies, patients who underwent LND compared to those who did not have LND had no significant differences in overall survival (HR: 1.08 (0.78-1.40 95% CI, p=.58). The I^2^ test (1.62) revealed no significant heterogeneity. Patients who underwent LND compared to those who did not showed no significant difference between the study groups (HR: 1.20 (0.94-1.54 95% CI, p=0.13). The I^2^ test (4.18) revealed no significant heterogeneity either. 

Esophageal Cancer 

The data from a single study showed there is no significant difference in overall survival between LND and no LND (CI: 0.85 (0.70- 1.04 95% CI, p = 0.11) and ELND vs. SLND (CI: 0.83 (0.71-0.97 95% CI, p = 0.14). Similar results were observed regarding recurrence-free survival (HR: 1.03 (0.93-1.14 95% CI, p= 0.57)). 

Discussion

Theoretically, LND performed for the purpose of staging can improve disease outcome, if there is a proven effective adjuvant treatment for the specific type of cancer. LND for staging purposes may be justified by assessing the risks of complications against the potential benefits of adjuvant therapy directed by pathologically proven lymph nodal metastasis [12, 13]. Increased operative time and other caveats. The therapeutic oncological benefit falls short when complications are taken into consideration [14-16]. 

LND has traditionally been used as a biomarker to guide further adjuvant treatment in high-risk patients. However, with the advent of evolving biomarkers, like molecular genetics, tumor markers, and advanced radiologic modalities, early stratification of an invasive disease is an achievable goal in the near future. Common examples are hormone-receptor status, triple-negative breast cancer (TNBC) in breast cancer, and Oncotype Dx in breast and prostate cancer [17,18]. In this new era of immunotherapy with fewer side effects and better treatment tolerance, limiting the surgical treatment to only standard dissection seems to be the better choice for patient care [19]. 

LND can potentially upstage malignancies by detecting microscopic metastatic disease. Adjuvant therapy based on pathology findings from LND can potentially shift patients between “good risk” and “poor risk” categories, allowing for non-treatment and treatment arms, respectively, and thereby improving outcomes in both arms. The detection of microscopic metastatic disease in the lymph nodes allows for the treatment of patients with low disease burden along with larger volumes of macroscopic lymph nodal disease, which could potentially contribute to improved outcomes in patients with lymph nodal metastatic disease. As a corollary, patients with clinically unsuspected lymph nodal metastatic disease would be enriched by removal of patients with microscopic metastatic disease, which would also contribute to improved clinical outcomes in the observation arm. The improved clinical outcomes following adjuvant treatment based on microscopic disease may be a result of selection bias euphemistically referred to as the Will Rogers phenomenon [20]. A proper randomization of patients is necessary to combat this selection bias and lack of improved therapeutic outcome in our analysis suggests that a similar selection bias following adjuvant therapy in patients with pathology-proven lymph nodal metastasis remains to be considered. 

A common attempt at improving the therapeutic benefit of LND is by increasing the extent of LND. In bladder cancer, performing ELND is associated with a higher incidence of lymphocele, while in breast cancer, axillary dissection can cause sensory neuropathy, lymphedema, and postoperative infection in the axilla [21, 22]. In prostate cancer, ELND is complicated by lymphocele, lower extremity edema, DVT, pelvic abscess, and ureteral injury [23-25]. ELND in general is associated with longer operative times, more blood loss, increased hospital stays, and a high post-op mortality rate. Therefore, the benefit of increasing the extent of LND needs to be considered in the context of increased complications and potential therapeutic benefit of adjuvant therapy. 

Our study is not without limitations. First, although we included only randomized controlled trials to minimize bias, the heterogeneity in surgical techniques, lymphadenectomy definitions, and perioperative practices across studies introduces variability that may impact outcome interpretation. Definitions of SLND and ELND varied by cancer type and study, and the number of lymph nodes removed was not uniformly reported, limiting our ability to perform granular comparisons. In addition, several trials included mixed-stage patient populations, and while we attempted to account for this in our selection criteria and analysis, unmeasured confounding due to disease stage may persist. The lack of consistent reporting on tumor biology, such as molecular subtypes or nodal burden, further limits our ability to identify subgroups that may benefit from LND. Finally, publication bias and the exclusion of non-English language studies may have led to the omission of relevant data. Despite these limitations, this meta-analysis provides a comprehensive and timely synthesis of high-quality evidence across multiple high-mortality solid organ malignancies. 

The main challenge we had was the difficulty of comparing a large spectrum of studies on multiple organ malignancies, because of heterogeneity between patients and treatment. At times, there were various LND groups; it was hard to control for surgical quality, and there was a risk of selection bias. The Will Rogers phenomenon refers to the “improved” survival of patients with cancer or other diseases by either reclassifying them into different prognostic groups, recognizing subtle disease manifestations, or using diagnostic modalities that allow the disease to be diagnosed at an earlier stage. The studies on LND are particularly susceptible to this selection and reclassification bias. Although randomization can minimize this bias, other challenges in conducting randomized surgical studies, such as the extent and quality of surgical dissection, often compound the biases. An important consideration in interpreting our findings is the potential influence of the Will Rogers phenomenon, a type of stage migration bias. This occurs when improvements in diagnostic sensitivity, such as the detection of microscopic lymph node metastases through LND, reclassify patients into more advanced disease categories without truly altering their prognosis. As a result, both the earlier and later stage groups may appear to have improved survival, even though overall outcomes remain unchanged. In the context of LND, this can lead to the illusion of benefit in both treated and untreated cohorts. Although randomized controlled trials can help mitigate this bias, their effects may still persist, particularly when pathological upstaging influences the administration of adjuvant therapy. The absence of survival benefit across multiple high-quality RCTs in our analysis suggests that even when stage migration occurs due to more extensive nodal evaluation, it may not translate into improved therapeutic outcomes. This underscores the need for future studies to disentangle the prognostic from the therapeutic role of LND, ideally incorporating molecular staging tools to refine risk stratification. 

There is strong evolving evidence that, despite different organs of origin, cancers in humans share common molecular and genetic events that determine clinical outcomes [26]. One of the commonalities is metastatic spread through the lymph nodes, which portends poorer clinical outcomes across all solid malignancies. While the value of LND in staging is clear, the therapeutic benefit is often debated, and efforts are made to improve the therapeutic benefit by extending the LND [27]. In prostate cancer, the therapeutic impact of ELND remains an area of active investigation, particularly given the typically prolonged natural history of the disease. Several randomized trials, including the study by Lestingi et al., have reported no significant difference in overall survival between ELND and SLND in intermediate- and high-risk patients. However, updated analyses with extended follow-up are beginning to emerge and may offer more definitive insight as survival events accumulate over time. It is important to recognize that in prostate cancer, overall survival may not be reached for many years, which can limit the interpretability of short- and mid-term outcomes. 

To our knowledge, this is the first combined systematic review and meta-analysis [28-58] on the role of LND in commonly diagnosed solid-organ malignancies. Our study does not demonstrate a therapeutic benefit for LND in any of the solid malignancies’ studies. 

## Conclusions

The findings of this meta-analysis have several important clinical implications. While LND, particularly ELND, may enhance staging accuracy, our data suggest that it does not consistently confer a survival advantage across commonly diagnosed solid organ malignancies. However, an exception was noted in breast cancer, where SLND was associated with improved overall survival compared to ELND. These findings support a more selective use of LND, prioritizing its role in guiding adjuvant therapy rather than pursuing it as a routine therapeutic intervention. Given the increased operative time, complication risk, and limited benefit observed with ELND, surgical planning should carefully balance oncologic goals with patient safety and quality of life. Future research should focus on identifying molecular and clinical subgroups that may derive therapeutic benefit from LND, and on integrating genomic risk classifiers and advanced imaging techniques to improve nodal staging. Prospective, organ-specific trials that stratify patients by tumor biology, disease stage, and treatment response are needed to clarify which patients, if any, truly benefit from more extensive nodal dissection. 

## Appendices

Appendix 1

Search Strategy for Systematic Review and Meta-Analysis

PubMed: The search strategy is below: 

(((((((randomized controlled trial[pt] OR controlled clinical trial[pt]) OR randomized[tiab]) OR placebo[tiab]) OR "clinical trials as topic"[MeSH Terms:noexp]) OR randomly[tiab]) OR trial[ti]) NOT ("animals"[MeSH Terms] NOT "humans"[MeSH Terms])) AND ((("lymph node excision"[MeSH Terms] OR lymph node excision[tw]) OR lymphadenectomy[tw]) AND (((((((((("breast neoplasms"[MeSH Terms] OR breast cancer[tw]) OR ("lung neoplasms"[MeSH Terms] OR lung cancer[tw])) OR ("liver neoplasms"[MeSH Terms] OR liver cancer[tw])) OR ("pancreatic neoplasms"[MeSH Terms] OR pancreatic cancer[tw])) OR ("prostatic neoplasms"[MeSH Terms] OR prostate cancer[tw])) OR ("urinary bladder neoplasms"[MeSH Terms] OR bladder cancer[tw])) OR ("endometrial neoplasms"[MeSH Terms] OR endometrial cancer[tw])) OR ("ovarian neoplasms"[MeSH Terms] OR ovarian cancer[tw])) OR ("esophageal neoplasms"[MeSH Terms] OR esophageal cancer[tw])) OR ("colorectal neoplasms"[MeSH Terms] OR colorectal cancer[tw]))) 

Embase: search strategy

Results 1,353 #16 #15 AND [embase]/lim NOT ([embase]/lim AND [medline]/lim) 3,556 #15 #13 AND #14 2,495,942 #14 'crossover procedure':de OR 'double-blind procedure':de OR 'randomized controlled trial':de OR 'single-blind procedure':de OR random*:de,ab,ti OR factorial*:de,ab,ti OR crossover*:de,ab,ti OR ((cross NEXT/1 over*):de,ab,ti) OR placebo*:de,ab,ti OR ((doubl* NEAR/1 blind*):de,ab,ti) OR ((singl* NEAR/1 blind*):de,ab,ti) OR assign*:de,ab,ti OR allocat*:de,ab,ti OR volunteer*:de,ab,ti 36,670 #13 #1 AND #12 1,732,688 #12 #2 OR #3 OR #4 OR #5 OR #6 OR #7 OR #8 OR #9 OR #10 OR #11 204,154 #11 'colorectal cancer'/exp OR 'colorectal cancer' 167,009 #10 'esophagus cancer'/exp OR 'esophagus cancer' 115,351 #9 'ovary cancer'/exp OR 'ovary cancer' 47,267 #8 'endometrium cancer'/exp OR 'endometrium cancer' 77,468 #7 'bladder cancer'/exp OR 'bladder cancer' 238,510 #6 'prostate cancer'/exp OR 'prostate cancer' 99,601 #5 'pancreas cancer'/exp OR 'pancreas cancer' 249,682 #4 'liver cancer'/exp OR 'liver cancer' 400,323 #3 'lung cancer'/exp OR 'lung cancer' 542,168 #2 'breast cancer'/exp OR 'breast cancer' 75,203 #1 'lymph node dissection'/exp OR 'lymphadenectoy'/kw 

This systematic review and meta-analysis followed PRISMA guidelines; however, the protocol was not registered in PROSPERO, which represents a limitation of this study.

## References

[1] Wu Y, Wang S, Huang Z (2001). Extent of lymphadenectomy in stage I-IIIA non-small cell lung cancer: a randomized clinical trial. Zhonghua Zhong Liu Za Zhi.

[2] Izbicki JR, Passlick B, Pantel K, Pichlmeier U, Hosch SB, Karg O, Thetter O (1998). Effectiveness of radical systematic mediastinal lymphadenectomy in patients with resectable non-small cell lung cancer: results of a prospective randomized trial. Ann Surg.

[3] Passlick B, Kubuschock B, Sienel W (2002). Mediastinal lymphadenectomy in non-small cell lung cancer: effectiveness in patients with or without nodal micrometastases - results of a preliminary study. Eur J Cardiothorac Surg.

[4] Sugi K, Nawata K, Fujita N (1998). Systematic lymph node dissection for clinically diagnosed peripheral non-small-cell lung cancer less than 2 cm in diameter. World J Surg.

[5] Darling GE, Allen MS, Decker PA (2011). Randomized trial of mediastinal lymph node sampling versus complete lymphadenectomy during pulmonary resection in the patient with N0 or N1 (less than hilar) non-small cell carcinoma: results of the American College of Surgery Oncology Group Z0030 Trial. J Thorac Cardiovasc Surg.

[6] Pharoah PD, Caldas C (1999). Molecular genetics and the assessment of human cancers. Expert Rev Mol Med.

[7] Sleeman JP, Thiele W (2009). Tumor metastasis and the lymphatic vasculature. Int J Cancer.

[8] Galimberti V, Cole BF, Zurrida S (2013). Axillary dissection versus no axillary dissection in patients with sentinel-node micrometastases (IBCSG 23-01): a phase 3 randomised controlled trial. Lancet Oncol.

[9] Roy P, Leizorovicz A, Villet R, Mercier C, Bobin JY (2018). Systematic versus sentinel-lymph-node-driven axillary-lymph-node dissection in clinically node-negative patients with operable breast cancer. Results of the GF-GS01 randomized trial. Breast Cancer Res Treat.

[10] Zavagno G, De Salvo GL, Scalco G (2008). A Randomized clinical trial on sentinel lymph node biopsy versus axillary lymph node dissection in breast cancer: results of the Sentinella/GIVOM trial. Ann Surg.

[11] Siegel RL, Miller KD, Fuchs HE, Jemal A (2022). Cancer statistics, 2022. CA Cancer J Clin.

[12] Colleoni M, Gelber S, Goldhirsch A (2006). Tamoxifen after adjuvant chemotherapy for premenopausal women with lymph node-positive breast cancer: International Breast Cancer Study Group Trial 13-93. J Clin Oncol.

[13] Al-Alao O, Mueller-Leonhard C, Kim SP (2020). Clinically node-positive (cN+) urothelial carcinoma of the bladder treated with chemotherapy and radical cystectomy: clinical outcomes and development of a postoperative risk stratification model. Urol Oncol.

[14] Sangha MS, Baker R, Ahmed M (2021). Axillary dissection versus axillary observation for low risk, clinically node-negative invasive breast cancer: a systematic review and meta-analysis. Breast Cancer.

[15] van Rijssen LB, Narwade P, van Huijgevoort NC (2016). Prognostic value of lymph node metastases detected during surgical exploration for pancreatic or periampullary cancer: a systematic review and meta-analysis. HPB (Oxford).

[16] García-Perdomo HA, Correa-Ochoa JJ, Contreras-García R, Daneshmand S (2018). Effectiveness of extended pelvic lymphadenectomy in the survival of prostate cancer: a systematic review and meta-analysis. Cent European J Urol.

[17] Knezevic D, Goddard AD, Natraj N (2013). Analytical validation of the Oncotype DX prostate cancer assay - a clinical RT-PCR assay optimized for prostate needle biopsies. BMC Genomics.

[18] Barzaman K, Karami J, Zarei Z (2020). Breast cancer: biology, biomarkers, and treatments. Int Immunopharmacol.

[19] Małkiewicz B, Kiełb P, Gurwin A, Knecht K, Wilk K, Dobruch J, Zdrojowy R (2021). The usefulness of lymphadenectomy in bladder cancer-current status. Medicina (Kaunas).

[20] Feinstein AR, Sosin DM, Wells CK (1985). The Will Rogers phenomenon. Stage migration and new diagnostic techniques as a source of misleading statistics for survival in cancer. N Engl J Med.

[21] Benedetti Panici P, Basile S, Maneschi F (2008). Systematic pelvic lymphadenectomy vs. no lymphadenectomy in early-stage endometrial carcinoma: randomized clinical trial. J Natl Cancer Inst.

[22] Gschwend JE, Heck MM, Lehmann J (2019). Extended versus limited lymph node dissection in bladder cancer patients undergoing radical cystectomy: survival results from a prospective, randomized trial. Eur Urol.

[23] Leissner J (2005). Lymphadenectomy for bladder cancer. Diagnostic and prognostic significance as well as therapeutic benefit [Article in German]. Urologe A.

[24] Omloo JM, Lagarde SM, Hulscher JB (2007). Extended transthoracic resection compared with limited transhiatal resection for adenocarcinoma of the mid/distal esophagus: five-year survival of a randomized clinical trial. Ann Surg.

[25] Huang X, Wang J, Chen Q, Jiang J (2014). Mediastinal lymph node dissection versus mediastinal lymph node sampling for early stage non-small cell lung cancer: a systematic review and meta-analysis. PLoS One.

[26] Shlyakhtina Y, Moran KL, Portal MM (2021). Genetic and non-genetic mechanisms underlying cancer evolution. Cancers (Basel).

[27] Neubauer NL, Lurain JR (2011). The role of lymphadenectomy in surgical staging of endometrial cancer. Int J Surg Oncol.

[28] Agresti R, Martelli G, Sandri M (2014). Axillary lymph node dissection versus no dissection in patients with T1N0 breast cancer: a randomized clinical trial (INT09/98). Cancer.

[29] Giuliano AE, Ballman KV, McCall L (2017). Effect of axillary dissection vs no axillary dissection on 10-year overall survival among women with invasive breast cancer and sentinel node metastasis: the ACOSOG Z0011 (Alliance) randomized clinical trial. JAMA.

[30] Krag DN, Anderson SJ, Julian TB (2010). Sentinel-lymph-node resection compared with conventional axillary-lymph-node dissection in clinically node-negative patients with breast cancer: overall survival findings from the NSABP B-32 randomised phase 3 trial. Lancet Oncol.

[31] Veronesi U, Viale G, Paganelli G (2010). Sentinel lymph node biopsy in breast cancer: ten-year results of a randomized controlled study. Ann Surg.

[32] Wetzig N, Gill PG, Espinoza D (2017). Sentinel-lymph-node-based management or routine axillary clearance? Five-year outcomes of the RACS Sentinel Node Biopsy versus Axillary Clearance (SNAC) 1 trial: assessment and incidence of true lymphedema. Ann Surg Oncol.

[33] Bonneau C, Bendifallah S, Reyal F, Rossi L, Rouzier R (2015). Association of the number of sentinel lymph nodes harvested with survival in breast cancer. Eur J Surg Oncol.

[34] Canavese G, Bruzzi P, Catturich A (2016). Sentinel lymph node biopsy versus axillary dissection in node-negative early-stage breast cancer: 15-year follow-up update of a randomized clinical trial. Ann Surg Oncol.

[35] Izbicki JR, Passlick B, Karg O (1995). Impact of radical systematic mediastinal lymphadenectomy on tumor staging in lung cancer. Ann Thorac Surg.

[36] Keller SM, Adak S, Wagner H (2000). A randomized trial of postoperative adjuvant therapy in patients with completely resected stage II or IIIA non-small-cell lung cancer. Eastern Cooperative Oncology Group. N Engl J Med.

[37] Wu Yl, Huang ZF, Wang SY, Yang XN, Ou W (2002). A randomized trial of systematic nodal dissection in resectable non-small cell lung cancer. Lung Cancer.

[38] Tentes AA, Mirelis C, Karanikiotis C, Korakianitis O (2007). Radical lymph node resection of the retroperitoneal area for left-sided colon cancer. Langenbecks Arch Surg.

[39] Fujita S, Mizusawa J, Kanemitsu Y (2017). Mesorectal excision with or without lateral lymph node dissection for clinical stage II/III lower rectal cancer (JCOG0212): a multicenter, randomized controlled, noninferiority trial. Ann Surg.

[40] Dell' Anna T, Signorelli M, Benedetti-Panici P (2012). Systematic lymphadenectomy in ovarian cancer at second-look surgery: a randomised clinical trial. Br J Cancer.

[41] Harter P, Sehouli J, Lorusso D (2019). A randomized trial of lymphadenectomy in patients with advanced ovarian neoplasms. N Engl J Med.

[42] Maggioni A, Benedetti Panici P, Dell'Anna T (2006). Randomised study of systematic lymphadenectomy in patients with epithelial ovarian cancer macroscopically confined to the pelvis. Br J Cancer.

[43] Panici PB, Maggioni A, Hacker N (2005). Systematic aortic and pelvic lymphadenectomy versus resection of bulky nodes only in optimally debulked advanced ovarian cancer: a randomized clinical trial. J Natl Cancer Inst.

[44] Volkmer A, Meier W, Fehm T (2019). Randomized phase III study on lymphadenectomy in advanced ovarian cancer [Article in German]. Der Onkologe (berlin).

[45] Kitchener H, Swart AM, Qian Q, Amos C, Parmar MK (2009). Efficacy of systematic pelvic lymphadenectomy in endometrial cancer (MRC ASTEC trial): a randomised study. Lancet.

[46] Wu X, Li B, Qiu J (2015). Hepatectomy versus hepatectomy with lymphadenectomy in hepatocellular carcinoma: a prospective, randomized controlled clinical trial. J Clin Gastroenterol.

[47] Hulscher JB, van Sandick JW, de Boer AG (2002). Extended transthoracic resection compared with limited transhiatal resection for adenocarcinoma of the esophagus. N Engl J Med.

[48] Zhang R, Ying K, Shi L, Zhang L, Zhou L (2013). Combined endobronchial and endoscopic ultrasound-guided fine needle aspiration for mediastinal lymph node staging of lung cancer: a meta-analysis. Eur J Cancer.

[49] Farnell MB, Pearson RK, Sarr MG (2005). A prospective randomized trial comparing standard pancreatoduodenectomy with pancreatoduodenectomy with extended lymphadenectomy in resectable pancreatic head adenocarcinoma. Surgery.

[50] Ignjatovic I, Knezevic S, Knezevic D (2017). Standard versus extended lymphadenectomy in radical surgical treatment for pancreatic head carcinoma. J BUON.

[51] Jang JY, Kang JS, Han Y (2017). Long-term outcomes and recurrence patterns of standard versus extended pancreatectomy for pancreatic head cancer: a multicenter prospective randomized controlled study. J Hepatobiliary Pancreat Sci.

[52] Nimura Y, Nagino M, Takao S (2012). Standard versus extended lymphadenectomy in radical pancreatoduodenectomy for ductal adenocarcinoma of the head of the pancreas: long-term results of a Japanese multicenter randomized controlled trial. J Hepatobiliary Pancreat Sci.

[53] Yeo CJ, Cameron JL, Lillemoe KD (2002). Pancreaticoduodenectomy with or without distal gastrectomy and extended retroperitoneal lymphadenectomy for periampullary adenocarcinoma, part 2: randomized controlled trial evaluating survival, morbidity, and mortality. Ann Surg.

[54] Pedrazzoli S, DiCarlo V, Dionigi R (1998). Standard versus extended lymphadenectomy associated with pancreatoduodenectomy in the surgical treatment of adenocarcinoma of the head of the pancreas: a multicenter, prospective, randomized study. Lymphadenectomy Study Group. Ann Surg.

[55] Nishihira T, Hirayama K, Mori S (1998). A prospective randomized trial of extended cervical and superior mediastinal lymphadenectomy for carcinoma of the thoracic esophagus. Am J Surg.

[56] Lestingi JF, Guglielmetti GB, Trinh QD (2021). Extended versus limited pelvic lymph node dissection during radical prostatectomy for intermediate- and high-risk prostate cancer: early oncological outcomes from a randomized phase 3 trial. Eur Urol.

[57] Touijer KA, Sjoberg DD, Benfante N (2021). Limited versus extended pelvic lymph node dissection for prostate cancer: a randomized clinical trial. Eur Urol Oncol.

[58] Luo C, Guo W, Yang J (2012). Comparison of mastoscopic and conventional axillary lymph node dissection in breast cancer: long-term results from a randomized, multicenter trial. Mayo Clin Proc.

